# Binding Energy Calculation of Patchouli Alcohol Isomer Cyclooxygenase Complexes Suggested as COX-1/COX-2 Selective Inhibitor

**DOI:** 10.1155/2014/850628

**Published:** 2014-11-17

**Authors:** Sentot Joko Raharjo, Chanif Mahdi, Nurdiana Nurdiana, Takheshi Kikuchi, Fatchiyah Fatchiyah

**Affiliations:** ^1^Biology Doctoral Program, Faculty of Science, Brawijaya University, Malang 65141, Indonesia; ^2^Academic of Pharmacy and Food Analysis, Putra Indonesia Malang, Malang 65123, Indonesia; ^3^Chemistry Department, Faculty of Science, Brawijaya University, Malang 65123, Indonesia; ^4^Medicine Pharmacology, Faculty of Medicine, Brawijaya University, Malang 65141, Indonesia; ^5^Graduate Life Science School, Ritsumeikan University, Biwako Kutsasu Campus, Kutsasu, Shiga 525-8577, Japan; ^6^Biology Department, Faculty of Science, Brawijaya University, Malang 65141, Indonesia

## Abstract

To understand the structural features that dictate the selectivity of the two isoforms of the prostaglandin H2 synthase (PGHS/COX), the three-dimensional (3D) structure of COX-1/COX-2 was assessed by means of binding energy calculation of virtual molecular dynamic with using ligand alpha-Patchouli alcohol isomers. Molecular interaction studies with COX-1 and COX-2 were done using the molecular docking tools by Hex 8.0. Interactions were further visualized by using Discovery Studio Client 3.5 software tool. The binding energy of molecular interaction was calculated by AMBER12 and Virtual Molecular Dynamic 1.9.1 software. The analysis of the alpha-Patchouli alcohol isomer compounds showed that all alpha-Patchouli alcohol isomers were suggested as inhibitor of COX-1 and COX-2. Collectively, the scoring binding energy calculation (with PBSA Model Solvent) of alpha-Patchouli alcohol isomer compounds (CID442384, CID6432585, CID3080622, CID10955174, and CID56928117) was suggested as candidate for a selective COX-1 inhibitor and CID521903 as nonselective COX-1/COX-2.

## 1. Introduction

Alpha-Patchouli alcohol was the major compound of Patchouli oil from Pogostemon Herba [1]. Quantitative approaches structure relationship Activity (QSAR) with molinspiration analysis showed that the alpha-Patchouli alcohol compounds have candidates as enzyme inhibitors, protein kinase inhibitors, and inhibitors of nuclear receptors [2]. Oral administration of Patchouli alcohol also increases protection against influenza virus infection in mice by increasing the immune response and attenuation of the systemic inflammatory response [3]. Furthermore, alpha-Patchouli alcohol has the effect of anti-inflammatory activity, by regulating the mRNA expression of the panel of inflammatory mediators, including TNF-*α*, IL-1*β*, iNOS, and COX-2 [4]. The PubChem databases for the alpha-Patchouli isomer were CID442384, CID521903, CID6432585, CID3080622, CID10955174, and CID56928117.

Cyclooxygenase (COX or prostaglandin H-synthase) is the enzyme responsible for the committed step in prostaglandin biosynthesis, generating the product prostaglandin-H2 (PGH2). Various prostaglandin synthases then convert PGH2 into several different prostaglandins and thromboxane. These prostaglandins and thromboxane target specific G protein-coupled receptors and play major roles in regulation of renal function, platelet aggregation, protection of the stomach lining, and numerous other homeostatic tasks, as well as mediation of the cellular inflammatory response. The former “housekeeping” functions are attributed to the first of two established COX isoforms, the constitutively expressed COX-1, while the inflammatory response is associated largely with the inducible isoform, COX-2. Inhibition of COX-2 produces the analgesic, antipyretic, and anti-inflammatory effects typical of nonsteroidal anti-inflammatory drugs (NSAIDs), while inhibition of COX-1 is responsible for the antithrombotic effects of aspirin and other nonselective NSAIDs, as well as many of their side effects, such as gastric ulcer formation. The many therapeutically useful effects of COX inhibition have made the NSAIDs among the most widely used drugs of the past century. Since selective COX-2 inhibition can provide analgesic and anti-inflammatory effects with reduced undesirable gastric side effects, COX-2 selective inhibitors such as celecoxib and rofecoxib have become some of the most widely used prescription medications in the developed world. However, recent reports that COX-2 selective inhibitors may increase the risk of heart attack in some patients have caused great concern and stimulated increased interest in these enzymes [5, 6]. COX-1 and COX-2 selectivity of NSAIDs were determined by the IC_50_ value and the selectivity index (SI). During this time, the determination of IC_50_ analysis was performed* in vitro* and* in vivo* by using some COXs activity detection methods: (1) oxygen uptake method; (2) peroxidase method; (3) enzyme immunoassay (EIA); (4) radioimmunological assay (RIA) [7]. This study is expected to further develop ligands NSAIDs as COX selective inhibitors based on* in silico* analysis.

We have assessed the benefit of a virtual screening of alpha-Patchouli alcohol isomer (CID521903, CID442384, and/or CID6432585) as inhibitors of cyclooxygenase (COX-1/COX-2) isoenzymes, only with interaction energy by hydrogen binding by LeadIT Biosolve software [8]. Interaction isomer of alpha-Patchouli alcohol (CID442384, CID521903, and CID6432585) with COX-1 using the rigid docking Hex 8.0. Furthermore, the validation docking using flexible docking LeadIT Biosolve software [2]. Results of previous* in silico* analysis also were predicting Patchouli oil compounds as candidate ligand receptor to using COX-1 and COX-2. Alpha-Patchouli alcohol isomers have ability as inhibitor COX-1 and COX-2. LeadIT Biosolve software was also equipped with a predictive scoring-free energy binding between the ligands and receptor. The analysis shows the ligand CID521903 as the best inhibitor selective for COX-2 [9]. The scoring energy by LeadIT Biosolve can never be more than a rough approximation of the free energy of binding, because the scoring energy was using a simple function based on a single configuration of a receptor-ligand complex [10]. LeadIT (FlexX) is a flexible docking method that uses an incremental construction (IC) algorithm and a pure empirical scoring function similar to the one developed by Böhm and coworkers to place ligands into the active site. IC algorithms first dissect each molecule into a set of rigid fragments according to rotatable bonds and then incrementally assemble the fragments around the binding pocket. The free binding energy Δ*G* of the protein-ligand complex is given by Δ*G* = Δ*G*_0_ + Δ*G*_*rot*⁡_ × *N*_*rot*⁡_ = Δ*G*_0_ + Δ*G*_lipo_ · ∑_lip ocount_ *f*∗(Δ*R*). Here, *f*(Δ*R*, Δ*α*) is a scaling function penalizing deviations from the ideal geometry. *N*_*rot*⁡_ is the number of free rotatable bonds that are immobilized in the complex. The terms Δ*G*_0_ are adjustable parameters. Δ*G*_lipo_ is lipophilic contact energy [11].

Improved methods are required to predict the position and orientation (poses) of binding to the target protein of low molecular weight compounds identified in fragment screening campaigns. This is particularly important to guide initial chemistry to generate structure activity relationships for the cases where a high resolution structure cannot be obtained. There have recently been significant developments in the use of implicit solvent methods in bimolecular simulations and estimation of binding affinity for protein, ligand, and complexes. Scoring schemes based on such methods have been employed in docking and virtual screening [12]. To further explore the structural characters of the COX-1/COX-2-alpha-Patchouli alcohol isomer complexes, molecular docking, molecular dynamics simulations, and MM-PBSA (molecular mechanical and poisson born/surface accessible) binding-free-energy calculations were performed on COX-1 and COX-2 systems in complexes with alpha-Patchouli alcohol isomers (CID442384, CID521903, CID6432585, CID3080622, CID10955174, and CID56928117). The purpose of our study is to calculate qualitatively binding energy COX-1/COX-2-alpha Patchouli alcohol isomer complexes as suggested COX-1/COX-2 inhibitor selective. Results of the study also support the use of Patchouli oil Pogostemon Herba as a potential therapeutic agent by targeting alpha-Patchouli alcohol and also help the development of novel COX-1/COX-2 inhibitors selective based on sesquiterpenoid and other natural products.

## 2. Materials and Methods

### 2.1. Ligand Preparation

Alpha-Patchouli alcohol isomers (CID442384, CID521903, CID6432585, CID3080622, CID10955174, and CID56928117) were downloaded from http://pubchem.ncbi.nlm.nih.gov/ as 3D-SDF format, and then its energy form was minimized and converted to 3D-PDB format by Open Babel 2.3.1 in Hex 8.0 as ligand for virtual screening.

### 2.2. COX Protein Receptor

3D model from PDB ID: 1PTH was obtained from SWISS-MODEL repository for cyclooxygenase-1 (COX-1) (http://www.rcsb.org/pdb/explore/explore.do?structureId=1pth) and 3D model from PDB ID: 6COX for cyclooxygenase-2 (COX-2) [13]. We use Ramachandran plot analysis for using validation protein receptor [14].

### 2.3. Docking of Ligand-Protein and Visualization

We used Hex8.0 software (http://hex.loria.fr) for rigid docking to compute possible interaction COX-1 and COX-2 with Alpha-patchouli alcohol isomers (CID442384, CID521903, CID6432585, CID3080622, CID10955174, and CID56928117) on the interaction site. Output of the docking was refined using Discovery Studio Client 3.5 software and AMBER12 software. We used Discovery Studio Client 3.5 to perform interaction; ligand binds to COX-1/COX-2 and Ramachandran plot analysis.

### 2.4. Virtual Molecular Dynamic and Binding Energy Calculation

We also use AMBER12 software and Virtual Molecular Dynamics 1.9.1 to simulate most possible native complex structure of alpha-Patchouli alcohol isomers (CID442384, CID521903, CID6432585, CID3080622, CID10955174, and CID56928117), respectively, that bind with COX-1 and COX-2 in molecular dynamic with Model Solvent of MM-PBSA (molecular mechanical and poisson born/surface accessible), which included both backbone and side-chains movements. Thereafter, we used AMBER12 to refine the candidate models according to a binding energy calculation for scoring of virtual screening alpha-Patchouli alcohol isomer compounds as inhibitor selective for COX-1 and/or COX-2. Molecular dynamics (MD) were carried out using AMBER12 and the AMBER-99 force field. The initial structure of the alpha-Patchouli alcohol inhibitor complex was taken for each compound from the Hex 8.0 docking study. The ligand force fields parameters were taken from the General Amber force Field (GAFF), whereas AM1 ESP atomic partial charges were assigned to the inhibitors. Prior to the free MD simulations, two steps of relaxation were carried out; in the first step, we kept the protein fixed with a constraint of 500 Kcal*·*mol^−1^ *·*Å^−1^. In the second step, the inhibitor structures were relaxed for 0.5 ps, during which the protein atoms were restrained to the X-ray coordinates with a force constant of 500 Kcal*·*mol^−1^ *·*Å^−1^. In the final step, all restraints were removed and the complexes were relaxed for 1 ps. The temperature of the relaxed system was then equilibrated at 300 Kelvin through 20 ps of MD using 2 fs time steps. A constant volume periodic boundary was set to equilibrate the temperature of the system by the Langevin dynamics using a collision frequency of 10 ps^−1^ and a velocity limit of 5 temperature units. During the temperature equilibration routine, the complex in the solvent box was restrained to the initial coordinates with a weak force constant of 10 Kcal*·*mol^−1^ *·*Å^−1^. The final coordinates of the temperature equilibration routine (after 20 ps) were then used to complete a 1 ns molecular dynamics routine using 2 fs time steps, during which the temperature was kept at 300 Kelvin. For the Langevin dynamics a collision frequency of 1 ps^−1^ and a velocity limit of 20 temperature units were used. The pressure of the solvated system was equilibrated at 1 bar at a certain density in a constant pressure periodic boundary by an isotropic pressure scaling method employing a pressure relaxation time of 2 ps. The time step of the free MD simulations was 2 fs with a cut-off of 9 Å for the nonbonded interaction, and SHAKE was employed to keep all bonds involving hydrogen atoms rigid. Calculation of binding energy is determined by the equation Δ*G* = *G*_complex_ − [*G*_protein_ + *G*_ligand_] [15, 16].

## 3. Result

### 3.1. Ligand Alpha-Patchouli Alcohol Isomers

**Figure 1:**
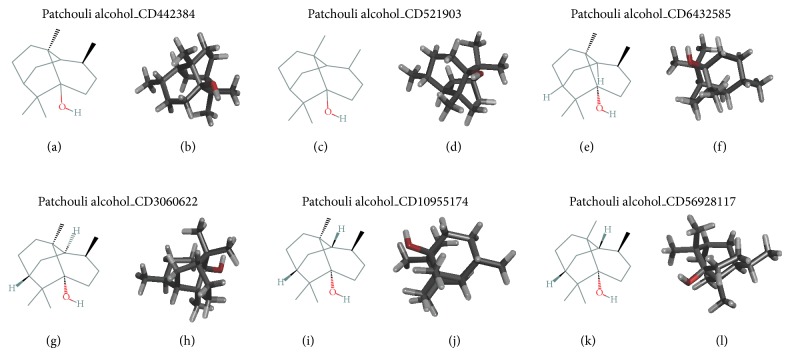
2D and 3D structure of alpha-Patchouli isomers (CID442384, CID521903, CID6432585, CID3080622, CID10955174, and CID56928117).

Ligand alpha-Patchouli alcohol was obtained from http://pubchem.ncbi.nlm.nih.gov/. Structure of 2D and 3D alpha-Patchouli alcohol has a several isomers: CID442384, CID521903, CID6432585, CID3080622, CID10955174, and CID56928117, as shown in Figure 1.

### 3.2. COX Protein Receptor

**Figure 2:**
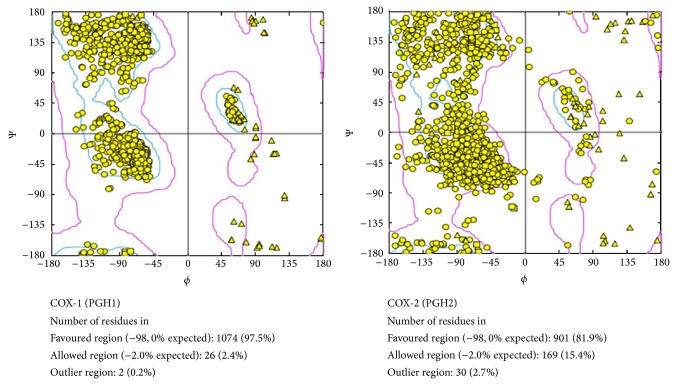
Ramachandran plot analysis of COX-1 and COX-2.

Both units of 3D structure protein isoforms COX-1 and COX-2 performed Ramachandran plot analysis, as presented Figure 2.

### 3.3. Docking of Ligand-Protein and Visualization

**Figure 3:**
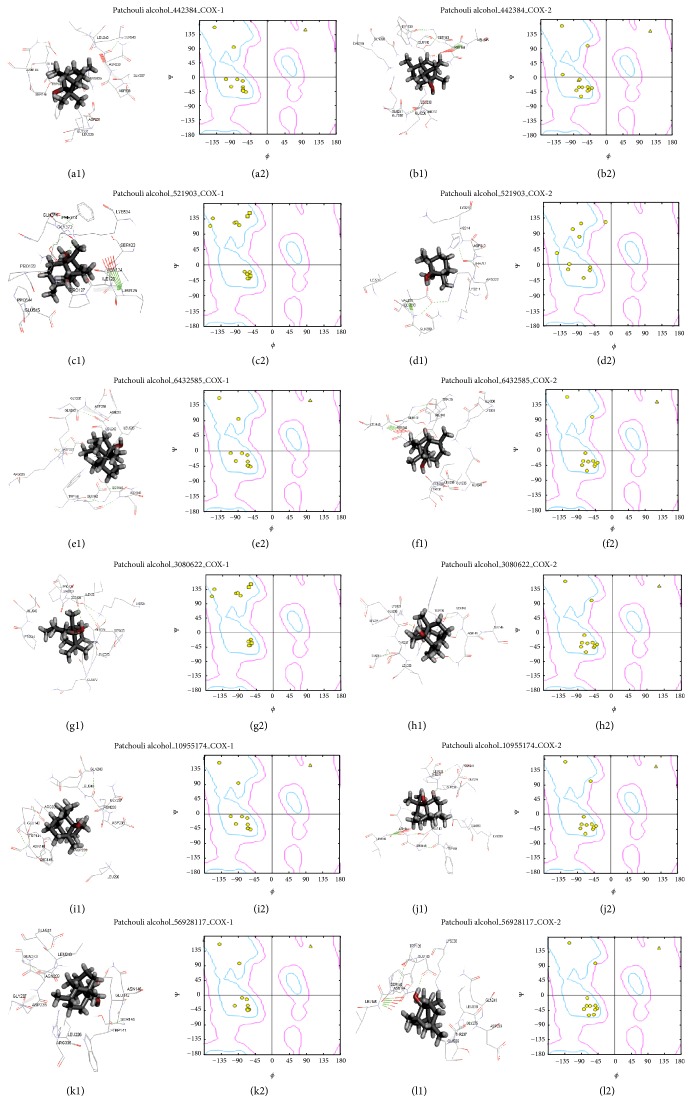
Modeling analyses suggest that alpha-Patchouli alcohol isomer binds to COX-1 and COX-2. (a1) to (l1) 3D active site structure of COX-1/COX-2-alpha-Patchouli alcohol isomers (CID 521903, CID6432585, CID442384, CID306022, CID10955174, and CID56928117) complexes; we used Hex 8.0 Docking Software and Discovery Studio 3.5 Viewer Software; (a2) to (l2) Ramachandran plot analysis of COX-1/COX-2-alpha-Patchouli alcohol (CID 521903, CID6432585, CID442384, CID306022, CID10955174, and CID56928117) complexes; we used Discovery Studio 3.5 Viewer Software.

Next step is docking (ligand to protein) alpha-patchouli isomers to COX-1 and COX-2 using rigid docking Hex 8.0 software. The results of the docking performed active visualization and Ramachandran plot analysis using Discovery Studio 3.5 software, as presented in Figure 3.

### 3.4. Virtual Molecular Dynamic and Binding Energy Calculation

**Figure 4:**
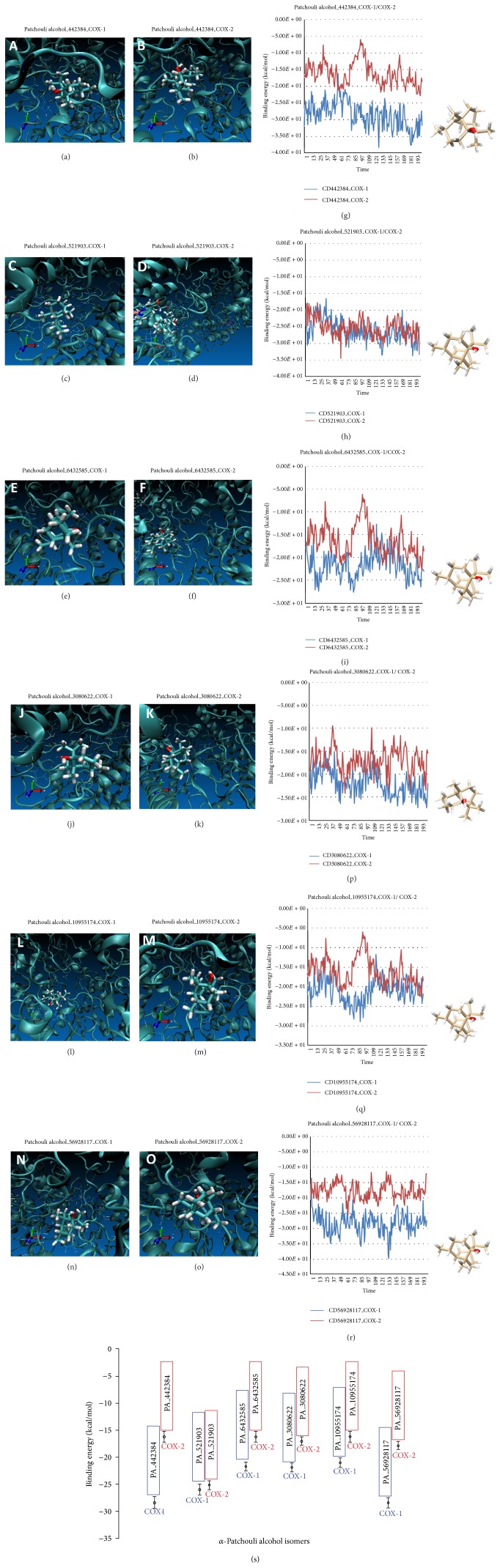
Binding energy calculation of alpha-Patchouli alcohol isomers binds to COX-1/COX-2. (a)–(f) and (j)–(o) Virtual Molecule Dynamic Complexes of COX-1/COX-2-alpha-Patchouli alcohol isomers. (g), (h), (i), (p), (q), and (r) Comparison of binding energy calculation of alpha-Patchouli alcohol isomer-COX-1 (blue) and COX-2 (red) complexes. (s) Histogram of Binding energy calculation of COX-1 (blue)/COX-2 (red)-alpha-Patchouli alcohol isomer complexes.

We were using AMBER12 software and Virtual Molecular Dynamics 1.9.1 to simulate the most possible native complex structure of alpha-Patchouli alcohol isomers (CID442384, CID521903, CID6432585, CID3080622, CID10955174, and CID56928117), respectively, that binds with COX-1 and COX-2 in molecular dynamic with MM-PBSA Model Solvent. The MD simulations of the alpha-Patchouli alcohol isomers-inhibitor complexes were shown in Figures 4(a)–4(f) and 4(j)– 4(o). We also acquire the results of the analysis of 200 poses: the complex energy, energy ligand protein, and energy. The binding energy was calculated use the following equation Δ*G* = *G*_complex_ − [*G*_protein_ + *G*_ligand_] [15, 16], as shown in Figures 4(g)–4(i), 4(p)–4(r), and 4(o). Analysis of the active site and the binding energies COX-1/COX-2-alpha-patchouli isomers are summarized and presented in Table 1.

## 4. Discussion

Alpha-Patchouli alcohol has molecular weight: 222.36634 g/mol; molecular formula: C_15_H_26_O; XLogP3-AA: 4.1; H-Bond Donor: 1; and H-Bond Acceptor: 1. The number of isomers of alpha-Patchouli alcohol is 6 isomers. In Figure 1, 2D and 3D alpha-Patchouli isomers (CID442384, CID521903, CID6432585, CID3080622, CID10955174, and CID56928117) show the different position of hydroxyl group and hydrogen atom. 3D structure of alpha-Patchouli alcohol obtained 3D-SDF format from http://pubchem.ncbi.nlm.nih.gov/. For the preparation of docking, 3D-SDF format of isomers was converted to 3D-PDB using open babel software. Open babel software was the software to convert molecular modeling data files. This program helps to search, convert, analyze, or store data which has a wide range of applications in the different fields of molecular modeling, computational chemistry, and so forth. For a common user, it helps to apply chemistry aspect without worrying about the low level details of chemical information. It also converts crystallographic file formats (CIF, ShelX), reaction formats (MDLRXN), molecular dynamics and docking (AutoDock, Amber), 3D viewers (Chem3D, Molden), and chemical kinetics and thermodynamics (ChemKin, Thermo) (http://www.openbabel.org).

Structurally, the two cyclooxygenase enzymes, COX-1 and COX-2, are homodimers of two 70 kDa subunits related by a C2 axis of symmetry. Isoenzymes of COX are enzymes that have a dimer of two identical subunits, and each subunit contains two separate active sites. Two small arms attach the enzyme strongly to the inner surface of smooth endoplasmic reticulum [17]. The crystallographic structures of cyclooxygenase have four functional domain. There are membrane binding domain, dimerization domain, catalytic domain and the open cleft of the peroxidase active site is observable at the top of each monomer [18]. Each monomer consists of a globular, catalytic domain, a membrane-binding domain, and an epidermal growth factor (EGF) type domain. COX-1 and COX-2 protein in dimeric form bound to lipid bilayer membranes. Molecular dynamics simulations within an aqueous environment on 25 ns with arachidonic acid were bound in the cyclooxygenase sites. This study reveals some key differences between the two isozymes that include the orientations at which they sit on the surface of the membranes and the depths to which they embed within the membranes. The differences in membrane association of the isozymes indicate that they may integrate distinctively with the same membrane and/or with different membranes or their lipid components. This results indicate that arachidonic acid can be bound in the cyclooxygenase active site in distinct catalytically competent conformations that lead to certain hydroperoxy acids, and the arachidonic acid and/or cyclooxygenase sites undergo a conformational change which makes only one subunit of each homodimer catalytically active [19]. The catalytic domain of each COX monomer has two distinct active sites, a cyclooxygenase site and a heme-containing peroxidase site [7]. The cyclooxygenase active site, which performs the first reaction in prostaglandin synthesis, is found deep within a pocket that opens into the membrane, allowing easy access to insoluble arachidonic acid. The peroxidase active site is on the upper surface of the enzyme [17, 18]. The cyclooxygenase site converts arachidonic acid (5,8,11,14-eicosatetraenoic acid or AA) to the hydroperoxy endoperoxide product prostaglandin-G2 (PGG2) via two highly region specific and stereoselective oxygen additions and two cyclization reactions. The generally accepted cyclooxygenase reaction mechanism involves generation of a tyrosyl radical at TYR-385 in the cyclooxygenase active site by the oxidized heme cofactor. This tyrosyl radical then stereospecifically attracts the C-13 PRO-S hydrogen of AA, forming a pentadienyl radical that extends from C-11 to C-15 and restoring the TYR-385 hydroxyl group. In the X-ray crystal structure of AA bound to COX-1, the AA C-13 PRO-S hydrogen atom is well positioned for radical attack by TYR-385, while the C-13 PRO-R hydrogen atom is fully shielded from TYR-385. NSAIDs commercial can be grouped, based on a chemical point of view, into three main classes: (1) carboxylic acids, (2) phenazones (pyrazolones, oxicams), and (3) nonacidic compounds. Salicylic acids and esters, acetic acids, propionic acids, and fenamic acids belong to “carboxylic acids” class. Similarly to arachidonic acid, most of NSAIDs of “carboxylic acids” class interacts with COX-1, forming a salt bridge with ARG-120 of the hydrophobic channel, which orientates the aromatic portion of these molecules in the direction of the TYR-385 at cyclooxygenase catalytic site, catalytic site. In turn, it was also essential for irreversible inhibition of COX-1 and COX-2 by aspirin. Aspirin covalently modifies both COX-1 and COX-2 through acetylation of SER-530 and was 10–100-fold more potent against COX-1 than against COX-2. The product of the reaction, salicylic acid, is also bound in the active site, which provides an explanation for the selective delivery of the acetyl group to SER-530. The salicylate carboxyl group formed an ion-pairs with ARG-120, which is located immediately below SER-530 [7].

Figure 2 shows the Ramachandran plot analysis of COX-1 and COX-2 protein receptor before rigid docking. It showed that COX-1 protein receptor had 97.5% favored regions, 2.4% allowed region, and, 0.2% outlier regions. Whereas, COX-2 protein receptor had 81.9% favored regions, 15.4% allowed region and 2.7% outlier regions. Ramachandran plot displays the main chain torsion angles phi, psi (*φ*, Ψ) (Ramachandran angles) in a protein of known structure. Verification of the built model was done to ensure whether the model was programmed correctly and the algorithms were implemented. Validation results determined that the distribution of amino acid residues was at the most favorable region in the Ramachandran plot. This is an indication of the stereochemical quality of the model taken for the structural analysis and also validated the target-ligand binding efficacy of the structure. The Ramachandran plot shows the phi-psi torsion angles for all residues in the structure (except those at the chain termini) which were classified according to their regions in the quadrangle [20]. The most favored regions are colored yellow, additional allowed/generously allowed region, and outlier regions are indicated as blue and pink fields, respectively.

The repeat rigid docking used Hex 8.0 software to compute possible interaction COX-1 and COX-2 with alpha-Patchouli alcohol isomers (CID442384, CID521903, CID6432585, CID3080622, CID10955174, and CID56928117) on its interaction site and the data are represented by Discovery Studio 3.5 software in (Figures 3(a1) to 3(l1)). The interaction site position of COX-1/COX-2-alpha-Patchouli alcohol complexes were analyzed using Discovery Studio3.5 Client software to get the Receptor-Ligand Interaction and Ramachandran plot, as shown in Figure 3. In Table 1 and Figure 3, the interactions active site of ligand alpha-Patchouli alcohol isomers with COX-1 and COX-2 protein receptor showed the differences in the position active site. The different positions are shown in the Ramachandran plot analysis and its amino acid residues in the receptor active site of COX-1 and COX-2, because of the differences position of hydrogen atoms and hydroxyl groups on each of the 3D isomers of alpha-Patchouli alcohol structure (Figure 1). The results of docking and analysis of the active site also show that all ligand alpha-Patchouli alcohol isomers are in the catalytic domain. Thus all the compounds have the capability of blocking oxygenated reaction and reaction peroxides; currently substrate arachidonic acid becomes PGH2. Each ligand, CID521903, was seen interacting with HEM682B group in COX-2-CID521903 complexes. This result proved that it would lead to inhibition of enzymatic reactions occurring COX-1 and COX-2. The analysis of active site showed that there are any difference and similarities of the active site of all ligand alpha-Patchouli alcohol isomers which is interact with receptor proteins COX-1 and COX-2. This difference is caused by different stereoisomers of hydrogen atoms and hydroxyl group in alpha-Patchouli alcohol isomers. The different position active site the complexes have led to interaction types, such as hydrogen bond, van der Waals, electrostatic and covalent bond. The different types of interactions in this complex will certainly affect its binding free energy.

The binding free energy calculation model solvent MM-PB/SA and MM-GB/SA methods are characterized by the use of Poisson-Boltzmann (PB) and Generalized Born (GB) models to compute the electrostatic component of the solvation free energy. The binding free energy of the protein-ligand complex is approximated by the following equation:

(1) $$\begin{array}{c} \Delta G=\Delta H-T\Delta S. \end{array}$$

*T* is the temperature of the system at 300 Kelvin. The free binding energy (Δ*G*_binds_) of the protein-ligand-complex were evaluated using MMPBSA (Molecular Mechanics-Poison Blotzmann Surface Area) method as implemented in AMBER12. MMPBSA has consistently been shown to be good method for comparing binding energies of similar ligands as it is case. MMPBSA computes the binding free energy by using a thermodynamic cycle that combines the molecular mechanical energy with the continuum solvent approaches [20]. The calculation of binding free energy is computed as

(2) $$\begin{array}{c} \Delta G=G_{\text{complex}}-\left[G_{\text{protein}}+G_{\text{ligand}}\right]. \end{array}$$

In (2), *G*_complex_ is the absolute free energy of the complex, *G*_protein_ is the absolute free energy of the protein, and *G*_ligand_ is the absolute free energy of the ligand [15, 16]. The free energy of each term was estimated as a sum of the three terms:

(3) $$\begin{array}{c} \left[G\right]=\left[E_{\text{MM}}\right]+\left[G_{\text{SOL}}\right]-T\cdot \left[S\right]. \end{array}$$

[*E*_MM_] is the molecular mechanics energy of the molecule expressed as the sum of the internal energy (bond, angle, and dihedral) (*E*_int⁡_), electrostatic energy (*E*_ele_), and van Der Waals term (*E*_vdw_):

(4) $$\begin{array}{c} \left[E_{\text{MM}}\right]=\left[E_{\mathrm{int}}\right]+\left[E_{\text{ele}}\right]+\left[E_{\text{vdw}}\right]. \end{array}$$

[*G*_SOL_] accounts for the solvation energy which can be divided into the polar and nonpolar part. The polar part accounts for the electrostatic contribution to solvation and is obtained by solving the linear Poisson Boltzmann equation in a continuum model of the solvent [20].

The stages of the binding energy calculation by AMBER12 include preparation, minimization, heating, and energy calculations (complex, protein, and ligand). We extracted 200 snapshots (at time intervals of 2 ps) for each species (complex, protein, and ligand). Furthermore, the visualization using Virtual Model Dynamic (VMD 1.9.1 Software) is shown in Figures 4(a)–4(f) and 4(j)–4(o), and then the binding energy calculation can be obtained from the data ligand energy, protein energy, and energy complex by AMBER12, 200 times/poses, respectively; next, the binding free energy calculation is calculated by (2) and shown in Figures 4(g), 4(h), 4(i), 4(p), 4(q), and 4(r) and summarized in Table 1.  Figure 4(s) shows that the binding energy calculation (PBSA Model Solvent) of COX-1_CID442384 complexes (−28.386 ± 1.102 Kcal*·*mol^−1^) was smaller than the COX-2_CID442384 complexes (−16.215 ± 0.985 Kcal*·*mol^−1^) and also ligands CID6432585, CID3080622, CID10955174, and CID56928117. The similar research, docking studies ligand salicin compound from* D. gangeticum* to COX-1 and COX-2 protein receptor, showed high binding affinity COX-2 protein (−5 Kcal/mol) and lesser interaction with COX-1 (−3.79 Kcal/mol). Therefore, salicin could predict as COX-2 inhibitor selective and anti-cancerous compound [21]. Collectively, our results suggest that alpha-Patchouli alcohol (CID442384, CID6432585, CID3080622, CID10955174, and CID56928117) was suggesting an inhibitor of COX-1 selective novelty. Figures 4(h) and 4(s) show the binding energy calculation of COX-1_CID521903 complexes similarity with COX-2_CID521903 complexes. Statistical analysis of* t*-test showed *t*_calculation_ = −2,583 < *t*_table_ = 2.823 (*df* = 0.01). The *E*_binds_ value of COX-1_CID521903 and COX-2_CID521903 complexes was no difference, so we suggested that CID521903 as non-selective inhibitor ligand of COX-1/COX-2.

**Figure 5:**
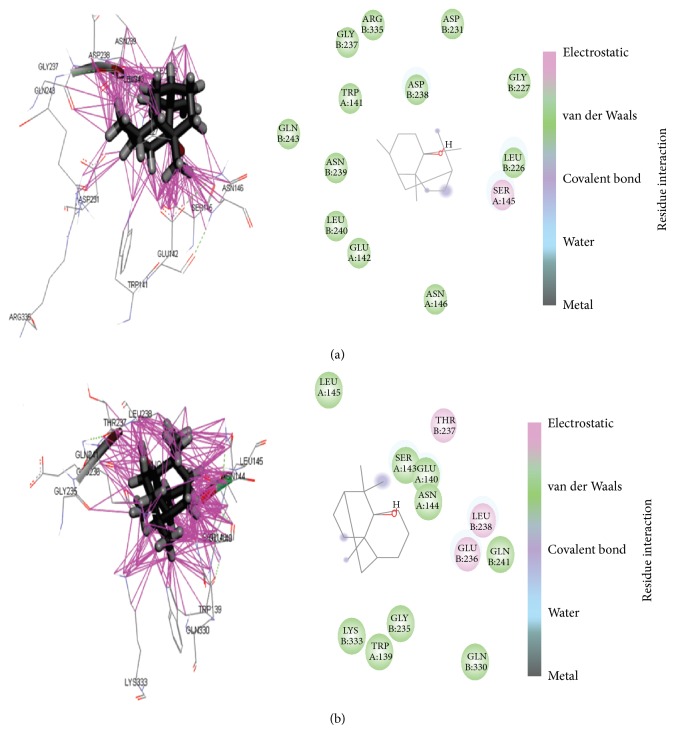
Ligand binding pattern and 2D interaction of CD442384 with COX-1 (a) and COX-2 (b).

Binding energy calculation is influenced by the position of the active ligand interactions of alpha-Patchouli alcohol and COX-1/COX-2 protein. The difference is the position of the active site also causes different types of interactions, such as hydrogen bonds (donor/acceptor), van der Waals interactions, covalent bond, and hydrophobic and aromatic interaction, as shown in Figure 5.

This interaction was affecting the type energy, such as potential/internal energy, electrostatic energy, and van der Waals energy, of each ligand energy, protein energy, and energy complex, respectively. And the binding energy calculation is Δ*G* = *G*_complex_ − [*G*_protein_ + *G*_ligand_]. The best scoring of difference binding energy calculation of alpha-Patchouli alcohol isomer COX-1 and COX-2 complexes was ligand CID442484. We conclude that ligand alpha-Patchouli alcohol CID442384 as the best inhibitors selective compound for COX-1. These* in silico* analysis data await conformation by IC_50_ value and the biological activity analysis.

## 5. Conclusion

Exploration of alpha-Patchouli alcohol isomer compounds as inhibitors of COX isoenzymes was alpha-Patchouli alcohol isomer compounds as development of group NSAIDs. Collectively, the scoring binding energy calculation (PBSA Model Solvent) of alpha-Patchouli alcohol compounds (CID442384, CID6432585, CID3080622, CID10955174, and CID56928117) was suggested as candidate for a selective COX-1 inhibitor and CID521903 as nonselective COX-1/COX-2.

## Figures and Tables

**Table 1:** Analysis of virtual modelling of COX-1/COX-2-alpha-Patchouli alcohol isomer complexes.

No.	Virtual modelling	Active site (by Hex 8.0 software and then Discovery Studio 3.5 software)		Binding energy calculation (Δ*E*_binding_ (Kcal/mol)) (Δ*E*_complex_ − Δ*E*_protein_ − Δ*E*_ligand_) Pose 200 by AMBER12 software		Suggestion
		COX-1	COX-2	COX-1	COX-2	
1	Alpha-Patchouli alcohol CID442384	TRP141A, GLU142A, SER145A, ASN146A, LEU226B, GLY227B, ASP231B, GLN243B, GLY237B, ASN239B, LEU240B, ASP238B, ARG335B	TRP139A, GLU140A, SER143A, ASN144A, LEU145A, GLY235B, GLU236B, THR237B, LEU238B, GLN241B, GLN330B	−28.386 ± 1.102 (VAR = 15.100, SD = 3.896, SE = 0.275)	−16.215 ± 0.985 (VAR = 12.056, SD = 3.481, SE = 0.246)	Selective of COX-1

2	Alpha-Patchouli alcohol CID521903	SER123A, ASN124A, LEU125A, ILE126A, PRO127A, SER128A, PRO129A, GLN372A, PHE373A, GLN274A, LYS534A, PRO544B, GLU545B	LYS211B, THR212B, ASP213B, HIS214B, LYS215B, ARG222B, ILE274B, GLN298B, GLU290B, VAL291B, HEM682B,	−25.995 ± 0.952 (VAR = 11.264, SD = 3.365, SE = 0.238)	−25.185 ± 0.815 (VAR = 8.255, SD = 2.880, SE = 0.204)	Nonselective

3	Alpha-Patchouli alcohol CID643285	TRP141A, GLU142A, SER145A, ASN146A, LEU226B, ASP231B, GLY237B, ASP238B, ASN239B, LEU240B, GLN243B, ARG335B	TRP139A, GLU140A, SER143A, ASN144A, LEU145A, THR237B, LEU238B, GLY235B, GLU236B, GLN241B, GLN330B, LYS333B	−21.724 ± 0.802 (VAR = 7.996, SD = 2.835, SE = 0.200)	−16.215 ± 0.985 (VAR = 12.056, SD = 3.481, SE = 0.246)	Selective of COX-1

4	Alpha-Patchouli alcohol CID3080622	SER123A, ASN124A, ILE126A, PRO127A, SER128A, PRO129A, GLN372A, PHE373A, GLN374A, LYS534A, PRO544B, GLU545B	TRP139A, SER143A, ASN144A, LEU145A, GLY235B, GLU236B, THR237B, LEU238B, GLN241B, LYS333B	−21.895 ± 0.770 (VAR = 7.367, SD = 2.721, SE = 0.192)	−17.020 ± 0.756 (VAR = 7.099, SD = 2.671, SE = 0.189)	Selective of COX-1

5	Alpha-Patchouli alcohol CID10955174	TRP141A, GLU142A, SER145A, ASN146A, LEU226B, ASP231B, GLY237B, ASP238B, ASN239B, LEU240B, GLN243B, ARG335B	TRP139A, GLU140A, SER143A, ASN144A, LEU145A, GLY235B, GLU236B, THR237B, LEU238B, GLN241B, GLN330B, LYS33B	−21.007 ± 0.859 (VAR = 9.177, SD = 3.037, SE = 0.215)	−16.215 ± 0.985 (VAR = 12.056, SD = 3.481, SE = 0.246)	Selective of COX-1

6	Alpha-Patchouli alcohol CID56928117	LEU226A, GLY237A, ASP238A, ASN239A, LEU240A, GLU241A, GLN243A, ARG335A, TRP141B, GLU142B, SER145B, ASN146B	GLY235A, GLU236A, THR237A, LEU238A, ASP239A, GLN241A, LYS333A, TRP139B, GLU140B, SER143B, ASN144B, LEU145B	−28.448 ± 0.955 (VAR = 11.339, SD = 3.376, SE = 0.239)	−17.833 ± 0.737 (VAR = 6.755, SD = 2.606, SE = 0.184)	Selective of COX-1
